# Vapour-Phase Selective Hydrogenation of γ-Valerolactone to 2-Methyltetrahydrofuran Biofuel over Silica-Supported Copper Catalysts

**DOI:** 10.3390/nano12193414

**Published:** 2022-09-29

**Authors:** Ramyakrishna Pothu, Prathap Challa, Rajendiran Rajesh, Rajender Boddula, Ravi Balaga, Putrakumar Balla, Vijayanand Perugopu, Ahmed Bahgat Radwan, Aboubakr M. Abdullah, Noora Al-Qahtani

**Affiliations:** 1 School of Physics and Electronics, College of Chemistry and Chemical Engineering, Hunan University, Changsha 410082, China; 2 Energy & Environmental Engineering Department, CSIR−Indian Institute of Chemical Technology, Hyderabad 500007, India; 3 Center for Advanced Materials (CAM), Qatar University, Doha 2713, Qatar

**Keywords:** biomass, 2-methyltetrahydrofuran, biofuel, hydrogenation, vapour-phase, selectivity

## Abstract

2-Methyltetrahydrofuran (MTHF) is a desirable biomass-based platform chemical with excellent potential as an ideal biofuel, green solvent, and raw material for synthesizing downstream chemicals. In this work, a series of copper nanoparticles encapsulated on SiO_2_ were prepared by the wet impregnation method and evaluated as efficient non-noble metal catalysts for the vapour-phase hydrogenation of γ-valerolactone (GVL) to MTHF in a fixed-bed reactor under mild reaction conditions. The obtained catalyst properties were determined by XRD, FE-SEM, TEM, UV-DRS, TPR, NH_3_-TPD, N_2_O decomposition and pore size distribution measurements. Meanwhile, the parameters/variables tuning their catalytic performance (activity, conversion, selectivity and stability) were examined. Various Cu loadings featured on the SiO_2_ support are essential for tuning the catalytic activity. Among the catalysts tested, a 5 wt% Cu/SiO_2_ catalyst showed a 97.2% MTHF selectivity with 71.9% GVL conversion, and showed a stability for 33 h time-on-stream, achieved at 260 °C and atmospheric pressure conditions. It was found that a huge dispersion of Cu metal in support, hydrogen activation ability, abundant acidic sites and surface area are all beneficial for improved MTHF selectivity.

## 1. Introduction

**Scheme 1:**
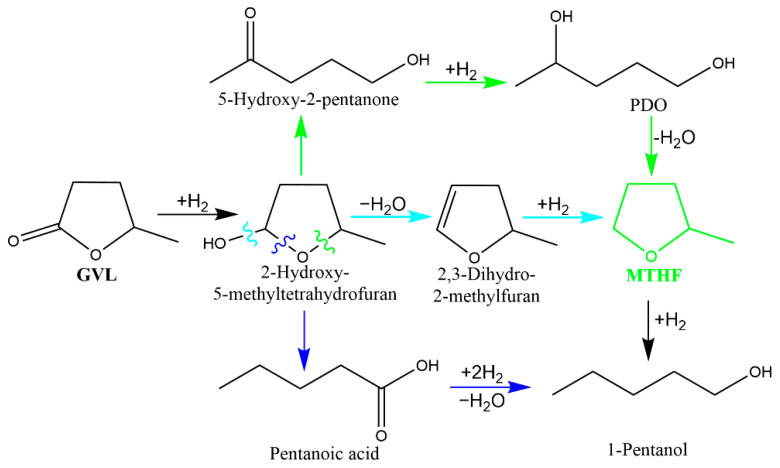
Possible routes of MTHF production from GVL over catalytic hydrogenation.

The majority of transportation fuels, chemicals, and materials originate from fossil fuels. As the worldwide need for energy grows, fossil fuels will be destroyed at an alarming rate. With the ever-worsening environment connected with the global fuel-intensive sector, extensive efforts are being made to find green alternative sustainable energy sources as the best methods for resolving problems [1]. Biomass is the most promising carbon-containing renewable and sustainable global resource, and it is typically used to replace fossil resources in the production of sustainable fuels and value-added chemicals such as 5-hydroxymethyfurfural (HMF), furfuryl alcohol, levulinic acid (LA), γ-valerolactone (GVL), and others [2,3]. The catalytic conversion of biomass-derived molecules into a broad range of fuels, chemicals and materials has piqued the interest of energy researchers all over the world in this context [2]. GVL has long been regarded as one of the most useful platform chemicals derived from biomass, with applications such as green solvents, flavoring agents, fuel additives, and polymer building blocks [3]. It can also be utilized as a starting material for the manufacture of alkanes and other important compounds. Value-added compounds, such as 1,4-pentanediol (PDO) and 2-methyltetrahydrofuran (MTHF), can be produced by hydrogenating GVL [4]. PDO is a valuable monomer for making bio-based polymers (polyesters), as well as an intermediary in the creation of perfumes, lubricants, and other chemicals [5]. MTHF is a promising biomass-based platform chemical with excellent potential as a green solvent [6,7], reagent for organometallic transformations [8] and biotransformations [9], and a viable biofuel or fuel additive due to its octane number of 87, which is comparable to gasoline, and as a P-series fuel component for the transportation infrastructure [10,11]. As a result, a competitive technique for producing 2-MTHF is necessary. Catalytic hydrogenation is one of the most effective and environmentally friendly ways to remove oxygen atoms from biomass and improve its practical values [12,13,14,15]. The acceptable route is thought to be involved in the production of MTHF from GVL as it undergoes the hydrodeoxygenation pathway. Many investigations have been conducted into the hydrogenation of LA to GVL and GVL to PDO, with a subsequent hydrogenation to MTHF and other compounds. Different catalyst qualities and reaction conditions would be required for each of these three steps, increasing the complexity of producing MTHF directly from LA. Only a few articles on GVL hydrogenation to yield MTHF or PDO have been identified. According to research, GVL hydrogenation can occur via two different mechanisms (Scheme 1).

The hydrogenolysis of GVL’s ester bond (O–(C=O)), followed by cyclodehydration of the PDO intermediate, yields MTHF. The other method includes ring-opening to hydrogenate GVL. Over bifunctional catalysts with metal and Bronsted sites, the breaking of the C–O bond on the methyl side of GVL and subsequent hydrogenation leads to the pentanoic acid formation, and with further hydrogenation forms 1-pentanol; both combinations result in pentyl valerates (valeric biofuels) [16,17,18]. On the basis of Au [19], Pt [20,21], Ru [22,23,24,25], Rh [18], Fe [26], and Co [27,28], catalytic selective hydrogenation of GVL to MTHF and/or PDO was carried out over homogeneous catalysts, with approximately 100% product yield. However, scarce noble metal catalysts are expensive and are inferior to heterogeneous catalysts due to the difficulty in isolating the catalyst from the reaction media, and so the cost of the catalysts can increase [22,23].

**Scheme 2:**
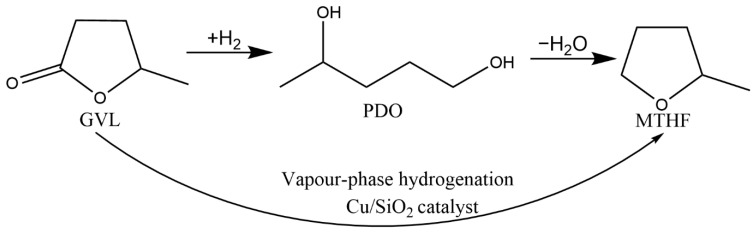
Selective conversion of GVL to MTHF over Cu/SiO_2_ catalyst.

The majority of recent research has been concentrated on Cu-based catalysts. Zhai et al. employed a Cu/MgO catalyst with a 9% Cu concentration composed through metal-organic chemical vapour deposition (MO-CVD) on a MgO support, which achieved a 64% yield of PDO at 240 °C in dioxane solvent and at 80 bar H_2_ [29]. Furthermore, it was discovered that the numerous basic sites of CuMg catalysts, through Cu minimal concentration, are required for the ring-opening/breaking of the furan ring in GVL to generate PDO [30]. A significant 96% yield was achieved using a catalyst that contained 30% Cu/ZrO_2_, annealed at 700 °C beneath reaction conditions (200 °C, 60 bar). On the other hand, 87% yield of MTHF was achieved by calcination with temperatures reduced to 300 °C and the reaction temperature raised to 240 °C [31]. Denise et al. used minimal amounts of Cu/SiO_2_ catalysts to hydrogenate GVL in cyclopentyl methyl ether solvent, yielding 78% PDO and no production of MTHF under reaction conditions (160 °C, 50 bar) [32]. Cu/SiO_2_ was synthesized using reduced Cu-hydroxosilicate and applied to GVL hydrogenation with n-butanol as the solvent in a fixed-bed reactor at 130 °C and 1.3 MPa H_2_, resulting in 32% GVL conversion and 67% PDO selectivity [33]. Despite its utility, these systems have several flaws, including cost, separation from product liquid, non-reusability, the need for precise handling of metal complexes, tedious work-up procedures, and the high price of catalyst preparation [13,22]. Vapor-phase reactions are more efficient and environmentally friendly than liquid-phase reactions because they require less H_2_ pressure, operate continuously, and allow for easy product separation [13]. Using a continuous flow, tubular, fixed-bed microreactor, we provided a vapour-phase catalytic hydrogenation pathway capable of selectively converting GVL to MTHF in the presence of silica-supported copper catalysts with hydrogen (Scheme 2). TPR, NH_3_-TPD, BET surface area, N_2_O decomposition, XRD, FE-SEM, and TEM were employed to characterize the catalysts, and the factors/parameters affecting their catalytic performance (activity, conversion, selectivity and stability) were deliberated.

## 2. Experimental Section

### 2.1. Catalyst Synthesis

A collection of Cu catalysts with altered Cu loadings, ranging from 2 to 20 (2, 5, 10, 15 and 20) wt% were synthesized by the wet-impregnation of copper nitrate trihydrate (Cu(NO_3_)_2_.3H_2_O, Sigma Aldrich, Germany) on the silica (SiO_2_, Sigma Aldrich, Germany) support. The catalysts were dried for 12 h at 110 °C and afterwards calcined for 5 h at 500 °C.

### 2.2. Catalyst Characterization

The XRD tests were carried out using a Rigaku MiniFlex diffractometer (Tokyo, TYO, Japan) using Cu Kα radiation that had been nickel filtered at 40 kV and 20 mA, with a ramp rate of 2°min^−1^. The textural properties of the silica-supported Cu materials were established using a Micromeritics ASAP 2020 system (Georgia, GA, USA) using N_2_ adsorption–desorption isotherms at 77 K. The catalysts were initially evacuated for 3 h at 200 °C. The BET equation was used to calculate the BET values. For the 200 kV conduction and TEM analysis, JEOL 2010 apparatus (Massachusetts, MA, USA) was used. After being sonicated for 10 min to dissolve the powder catalysts in an ethanol solution, the mixture was diffused onto Cu grids, and the microscope column received the sample holder. UV–Vis diffuse reflectance spectra (UV–DRS) analysis was carried out using a GBC UV–Visible Cintra instrument (Victoria, VIC, Australia). Using the 2920 Micromeritics device (Georgia, GA, USA), temperature-programmed desorption of ammonia (NH_3_-TPD) and temperature-programmed reduction of hydrogen (H_2_-TPR) analysis was used to examine the acidity of the catalysts. Typically, 0.1 g of material was prepared in an H_2_-TPR experiment by being exposed to He gas at 200 °C for 2 h, before being cooled to room temperature. TPR analysis was carried out between 25 °C and 600 °C with a ramping rate of 10 °C/min and a flow of 5 vol% H_2_/Ar. For the TPD run, the catalysts were pretreated, purged with He 50 mL for 1 h at 200 °C and the temperature was then lowered. The catalyst was treated with He 50 mL for 30 min at the same temperature after being reduced in situ using 5% H_2_-Ar in 40 mL for 2 h at 250 °C. Following that, 75 mL of 10% NH_3_-He gas underwent NH_3_ absorption for 1 h at 80 °C, followed by a 2 h purge at 120 °C for NH_3_ physisorption. The TPD run was carried out between 120 °C and 750 °C, and TCD assessed the NH_3_ output. The Brunauer–Emmett–Teller (BET) and Barret–Joyner-Halenda (BJH) methods were employed to measure the surface area and pore size. Images from scanning electron microscopy (SEM) were captured using a 20 kV-operated Philips/FEI Quanta 200F SEM (Texas, TX, USA).

### 2.3. Catalytic Reaction

Under typical conditions of 260 °C and 0.1 MPa of H_2_ pressure, the vapour-phase hydrogenation of GVL to MTHF synthesis and the process was evaluated in a continuous fixed-bed stainless steel tube reactor. The Cu/SiO_2_ catalyst was added to the reactor in amounts of around 0.4 g. The sample was initially reduced with 50 mL of H_2_ flow at 300 °C. The reactant feed was introduced using an HPLC pump, feed gas was used to pressurise the reactor, and the desired temperature was attained. Then 10% of the aqueous GVL feed was heated to 260 °C and subjected to an H_2_ flow, while being attached to the reactor intake. At various reaction conditions, the Cu/SiO_2_ catalytic performance was tested. A DB-wax column in a gas chromatograph was used to examine the products, which were collected hourly using an ice-water trap. The samples were diluted with methanol prior to the GC analysis, and an HP-1MS column was used for the GC-MS analysis. The conversion of GVL and selectivity of MTHF and PDO products were obtained using the equation below.

$$Conversion\;of\;GVL\;\left(\%\right)=\frac{\mathrm{GVL}\;\mathrm{moles}\;\left(\mathrm{in}\right)-\mathrm{GVL}\;\mathrm{moles}\;\left(\mathrm{out}\right)}{\mathrm{GVL}\;\mathrm{moles}\;\left(\mathrm{in}\right)}\times 100$$

$$Selectivity\;\left(\%\right)=\frac{\mathrm{moles}\;\mathrm{of}\;\mathrm{one}\;\mathrm{product}}{\mathrm{moles}\;\mathrm{of}\;\mathrm{all}\;\mathrm{products}}\times 100$$

## 3. Results and Discussion

### 3.1. Structure and Composition of the Catalysts

#### 3.1.1. XRD

**Figure 1:**
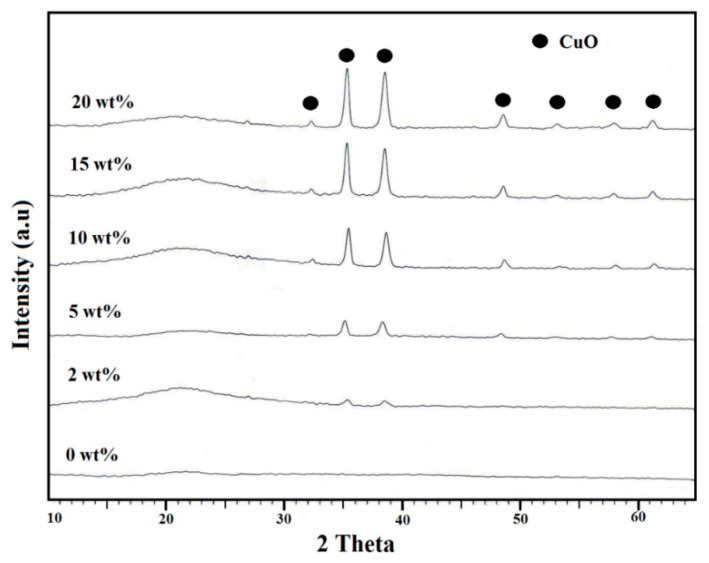
XRD patterns of various Cu/SiO_2_ catalysts.

Figure 1 depicts a variety of Cu/SiO_2_ catalysts where the CuO crystal structure on the SiO_2_ support was examined using XRD studies. It was clear that the silica support was in the amorphous condition and could not be seen by XRD because there is no peak corresponding to it. Only the characteristics peaks for the CuO crystalline phase were visible in all catalysts, as shown in Figure 1, which is consistent with a database of conventional CuO (JCPDS No. 80-1268) [34,35]. It is important to note that the peak intensity of CuO was seen to augment with increasing Cu% filling, indicating that increasing Cu% filling in the catalyst promotes the formation of CuO crystallites [34,35,36]. The observed XRD pattern does not contain any peaks associated with any other CuO phase.

**Figure 2:**
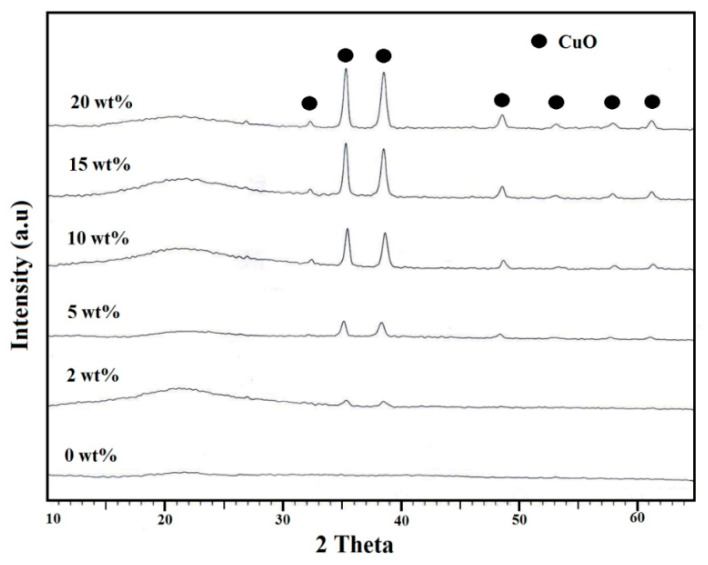
XRD patterns of series of reduced Cu/SiO_2_ catalysts.

Figure 2 displays the catalysts’ XRD patterns following reduction. All catalysts ascribed to metallic Cu were reported to have the two primary diffraction peaks at 2*θ* = 43.5° and 50.5°. Metallic Cu peak intensity grew as the amount of Cu was loaded, indicating that the amount of Cu(0) crystallinity also increased when the amount of Cu was loaded. Additionally, the CuO peaks were vanishing in the XRD pattern following the reduction of the series of Cu/SiO_2_ catalysts, showing that CuO had totally reduced to metallic Cu.

#### 3.1.2. BET surface Area

Nitrogen physisorption measurements were utilized to survey the textural characteristics (Table 1) of the various Cu/SiO_2_ catalysts. The specific surface area and cumulative pore volume of the pristine SiO_2_ samples were determined to be 346 m^2^ g^−1^ and 0.72 cm^3^ g^−1^, respectively. A small reduction in surface area was seen after Cu was impregnated into the surface of the SiO_2_ support. Table 1 shows that when the Cu loading increased, the catalyst surface area and pore volume dropped. This might be because CuO particles have filled the pores, as suggested by the XRD analysis.

#### 3.1.3. H_2_-TPR

**Figure 3:**
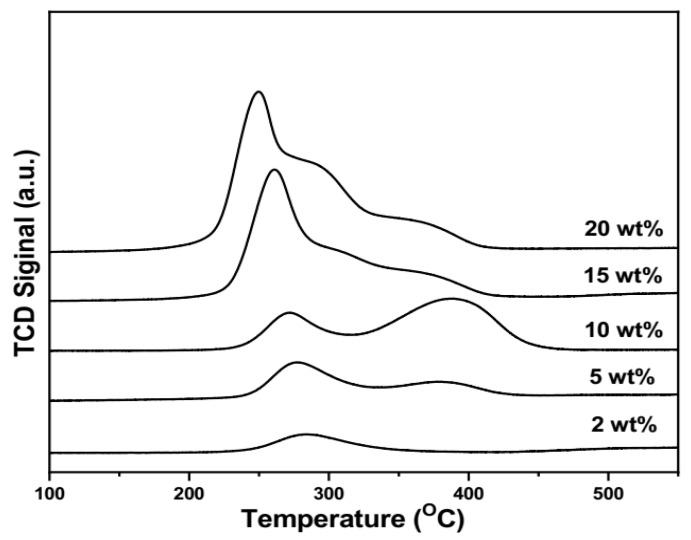
H_2_-TPR profiles of various Cu/SiO_2_ catalysts.

TPR examined the CuO species reducibility behaviour in various Cu/SiO_2_ catalysts. Figure 3 displays the TPR profiles of Cu/SiO_2_ catalysts with various Cu loadings, and Table 2 lists the outcomes of hydrogen uptake.

Low Cu loading (2 wt%) in the Cu/SiO_2_ catalyst had a single broad peak about 280 °C, which may be attributed to the reduction of isolated Cu species, as can be shown in the H_2_-TPR profiles. Another reduction peak, corresponding to the reduction of CuO crystallites to metallic Cu, arose at a high-temperature region (380 °C), when the Cu loading increased from 2 to 5 wt% [37]. While the high-temperature-peak intensity reached its highest value for the 10 wt% Cu loading in Cu/SiO_2_, and at a lower temperature region, the peak intensity clearly increased with increasing Cu%, from 2 to 20 wt%. Furthermore, the H_2_-TPR profile showed another reduction peak when the Cu loading was increased above 10 wt%. However, the peak intensity drastically reduced for samples with Cu loadings greater than 10 wt% at the higher temperature region (380 °C), indicating a weak interaction between Cu and silica [38]. With an increase in the sample’s Cu concentration, the value of hydrogen uptake rises. H_2_-TPR profiles of various Cu/SiO_2_ samples demonstrated the steady shift in the highest intensity peak (reduced CuO) at the lower temperature region when the Cu% loading was increased. This downward movement over the spinel phase of the Cu/SiO_2_, which reduced the interaction of the of the Cu^2+^ with Si^4+^ ions, can be attributed to the segregation of CuO [39].

**Figure 4:**
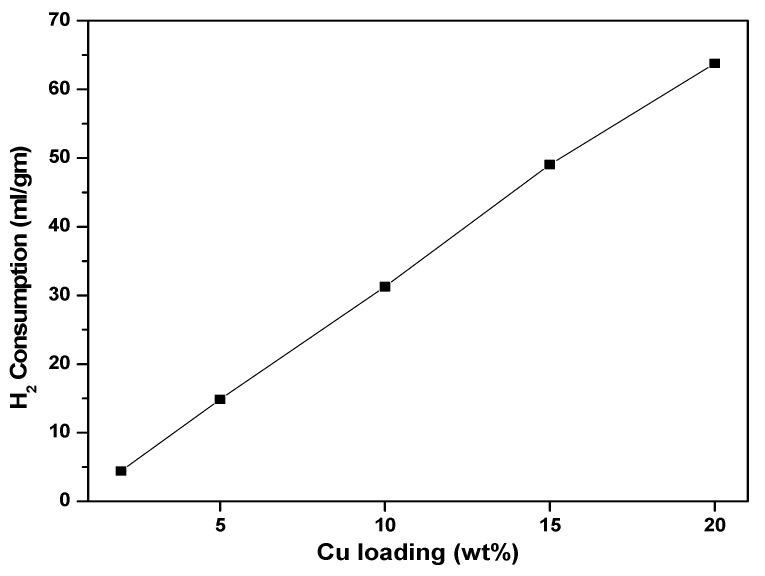
H_2_ consumed against Cu loading.

Figure 4 represents the quantity of hydrogen uptake determined from the H_2_-TPR profile versus Cu% loading. It was discovered that the overall hydrogen consumption matched the quantity needed for the complete conversion of CuO to metallic Cu. As a result, this result provided strong support for the XRD results.

#### 3.1.4. UV–Vis DRS

**Figure 5:**
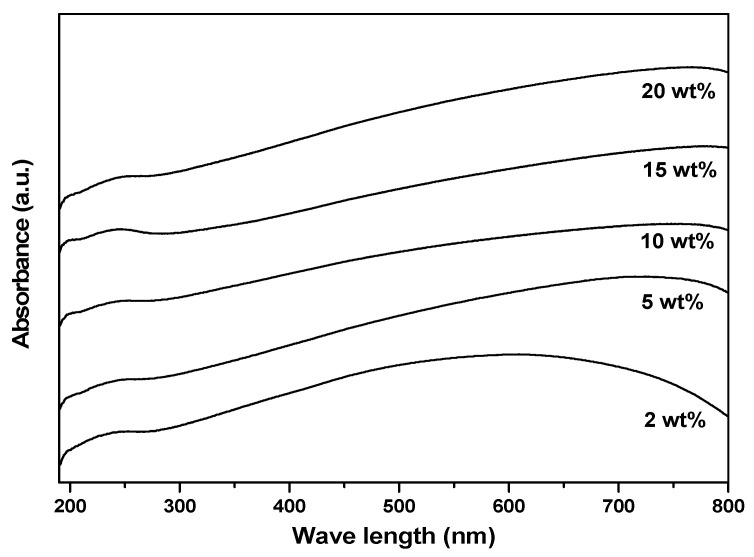
UV–Vis DRS profiles of various Cu/SiO_2_ catalysts.

The electronic state of different Cu/SiO_2_ catalysts can be studied using electronic spectroscopy in the UV–visible range (Figure 5). One band at approximately 200–250 nm and a broad absorption band at 350–800 nm were fitted to the spectra. In mononuclear or isolated species, O^2−^ to Cu^2+^ ligand-to-metal charge-transfer causes the first maximum in UV–Vis DRS spectra at 235 nm [40]. With an increase in Cu loading, this band was somewhat moved to a higher-wavelength region. The wide absorption band at 525 nm is due to Cu 2p electron d-d transitions in octahedral Cu particles which are surrounded by oxygen [41,42]. With an increasing Cu loading, this broad charge transfer band’s strength declines, and its position also appeared to migrate towards longer wavelengths.

#### 3.1.5. N_2_O decomposition

The Cu percentage dispersion in SiO_2_ support, Cu metal specific surface area (m^2^/g Cu), and Cu metal crystallize size (nm) were calculated using the N_2_O decomposition method (Table 3). According to Table 3, there is a maximum value for the dispersion and specific copper surface area for the 5 wt% Cu supported on an SiO_2_ sample. Cu surface area dropped to 34 m^2^/g_Cu_ when the Cu filling rose from 2 to 20 wt%. This finding indicated that increasing the Cu fiilling on the SiO_2_ support did not increase the number of Cu species shown. In fact, it actually prevented Cu species from spreading over the support. The accumulation of Cu clusters with a strong development in three dimensions, as evidenced by the increase in average size of Cu crystallites. As a result, an increased Cu loading on the SiO_2_ support reduced the Cu dispersion. These data are in excellent consistency with the TPR and XRD findings.

#### 3.1.6. SEM

**Figure 6:**
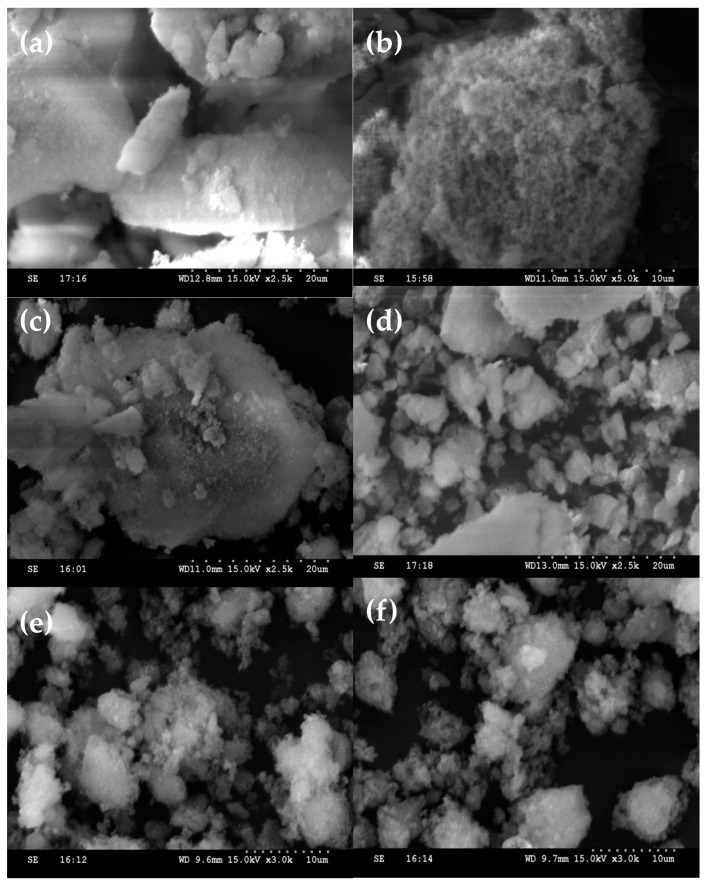
SEM images of pristine SiO_2_ (**a**), 2 wt% (**b**), 5 wt% (**c**), 10 wt% (**d**), 15 wt% (**e**), 20 wt% (**f**) of Cu/SiO_2_ catalysts.

SEM was used to examine the morphology of several Cu/SiO_2_ catalysts, as well as pure SiO_2_ supports. Figure 6 displays the SEM images corresponding to various Cu loadings. The creation of three-dimensional CuO phase structures for the supported catalyst is clearly seen in the various Cu/SiO_2_ catalysts. Due to the development of Cu crystallites in all samples, it was expected that there would be no monolayer-like dispersion. From 10 wt% Cu loadings, the development of CuO accumulation into three-dimensional structures became increasingly apparent. According to XRD and TPR studies, a heavy Cu% filling causes poor dispersion because the crystallites become larger and take on a three-dimensional shape.

#### 3.1.7. TEM

**Figure 7:**
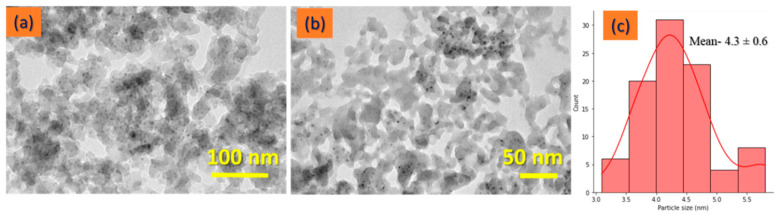
(**a**,**b**) TEM images of 5 wt% Cu/SiO_2_ catalyst with different resolution and (**c**) average particle size.

To further examine the microstructure of the best catalyst, images of the 5 wt% Cu/SiO_2_ catalyst were taken with different resolutions of TEM. TEM is a crucial technique in providing details regarding the size distribution, morphology, and particle size of Cu particles on the SiO_2_. Size distribution histograms obtained from TEM images and their particle size analysis, which counted extent 200 particles in each image, are displayed in Figure 7. The average Cu crystallite size of the reduced 5 wt% Cu/SiO_2_ catalyst was found to be 4.3 ± 0.6 nm. This implied that the Cu species active component is uniformly dispersed over the SiO_2_ support. This result is well-matched to the N_2_O decomposition results. This demonstrates clearly that CuO exists in a uniformly dispersed condition (Figure 6 and Figure 7) and that average Cu crystallites are less at lower Cu% loadings (Table 3) [43].

#### 3.1.8. NH_3_–TPD

**Figure 8:**
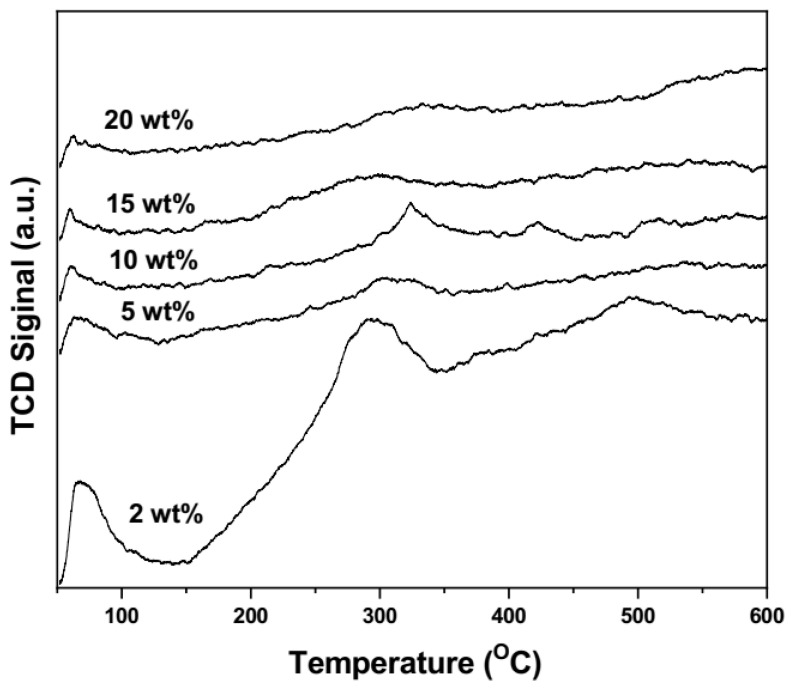
TPD profiles of various Cu/SiO_2_ catalysts.

NH_3_–TPD measurements were utilized to survey the surface acidity of the various Cu/SiO_2_ catalysts (Figure 8). Table 4 provides a summary of the total acidity in different Cu/SiO_2_ catalysts, determined from the peak area. The potency and quantity of the catalyst’s acidic sites are shown by the peak temperature and the amount of desorbed NH_3_, respectively. The desorption of ammonia bound to weak acid sites and physisorbed ammonia molecules was primarily responsible for the low-temperature peak in the ammonia TPD profiles, which is centred at 60–80 °C. The desorption of NH_3_ from moderate acid sites was responsible for the high-temperature desorption peak which was centred at 250–300 °C. The weak acid sites of the pristine SiO_2_ sample (40 µmol/g) showed a low-temperature peak at 74 °C and the moderate acid sites (192 µmol/g) showed a low-temperature peak at 297 °C, i.e., the total overall acidity of the pristine SiO_2_ was 232 µmol/g. Increased Cu loadings were observed to be inferior, as shown by the low-temperature desorption peak of NH_3_-TPD profile, because the catalyst surface area was reduced. On the other hand, the high-temperature NH_3_ desorbed peak is most pronounced at low Cu loadings, and gradually diminishes as Cu loading increases, suggesting a loss of adsorption sites caused by the agglomeration of Cu ions with the development of tiny CuO crystallites. Increasing Cu loading on the support resulted in a decrease in the overall acidity of the Cu/SiO_2_ catalysts. The highest amount of acidic strength was seen in the 5 wt% Cu/SiO_2_ catalyst, which is advantageous for the greater conversion activity of GVL to MTHF.

### 3.2. Catalytic Activity

#### 3.2.1. Influence of Cu Loading on SiO_2_ Support

We preliminarily investigated the GVL hydrogenation over various Cu/SiO_2_ catalysts by looking at the effect of metal loading on catalytic performance, as portrayed in Table 5. GVL conversion increased when Cu loading increased from 2 wt% to 5 wt%; it later decreased when the Cu% loading rose from 10 to 20 wt% at 200 °C and ambient pressure conditions. These findings unequivocally demonstrated that the reaction rate is significantly influenced by the acid concentration. The best and optimized results can be obtained using the 5 wt% Cu/SiO_2_ catalyst, which exhibited GVL conversion and MTHF selectivity as 21.6% and 98.1%, respectively. Due to the high diffusivity of Cu species on the SiO_2_ surface, favorable conditions for the maximum catalytic activity of 5 wt% Cu/SiO_2_ were created, with good dispersion of Cu, smaller particle sizes and strong acidic site availability, as confirmed by BET surface area, N_2_O decomposition and NH_3_-TPD studies.

The decline in the Cu-specific surface area and its corresponding surface acidity may be tentatively responsible for the drop-in activity with the increase in Cu content from 10 to 20 wt%. It should be noted that because the support had a smaller surface area, the multilayer deposition of active sites on the support prevents a higher Cu loading from necessarily leading to higher activities. According to these findings, the GVL conversion and selectivity were directly correlated with the acid concentration and the active Cu sites of the catalyst.

#### 3.2.2. Influence of TOF

**Figure 9:**
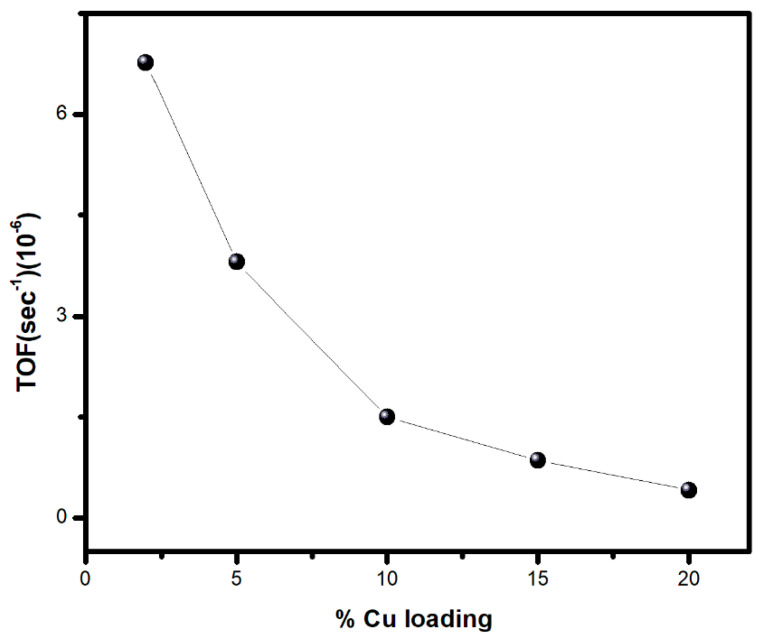
TOF vs. Cu% loading in different Cu/SiO_2_ catalysts.

The relationship between Cu loading and TOF is displayed in Figure 9, which can be used to determine the relationship between the hydrogenation of GVL and the amount of Cu loading. The rate of GVL molecules transformed per unit of time per exposed Cu site is known as TOF. Cu dispersion and hydrogenation activity are suggested to have a structure–activity link in Figure 9. Because of the CuO crystallite growth on the surface of the SiO_2_ support, it was discovered that the conversion decreased as the CuO loading was increased up to 20 wt%.

As shown in Table 6, the decline in the selectivity of GVL is presumably because of reduced dispersion and the greater particle size of Cu, which is evidently shown through the decomposition of N_2_O and XRD tests. The findings in Table 6 also imply a strong relationship between the catalyst’s activity and the N_2_O decomposition data.

#### 3.2.3. Influence of Reaction Temperature

The results of the investigation into the effects of reaction temperature on the hydrogenation reaction of GVL by the 5 wt% Cu/SiO_2_ catalyst in the 200–300 °C temperature range are summarised in Table 7. With an increase in reaction temperature from 200 to 300 °C, GVL conversion increases. A lack of sufficient energy for GVL dehydration into MTHF could be the cause of the low conversion seen at lower temperatures. The selectivity of MTHF drops from 91% to 84% when the reaction temperature rises from 200 to 300 °C. On the other hand, because the dehydration product of GVL has taken over, reaction temperatures beyond 260 °C are undesirable for this reaction.

#### 3.2.4. Influence of WHSV

**Figure 10:**
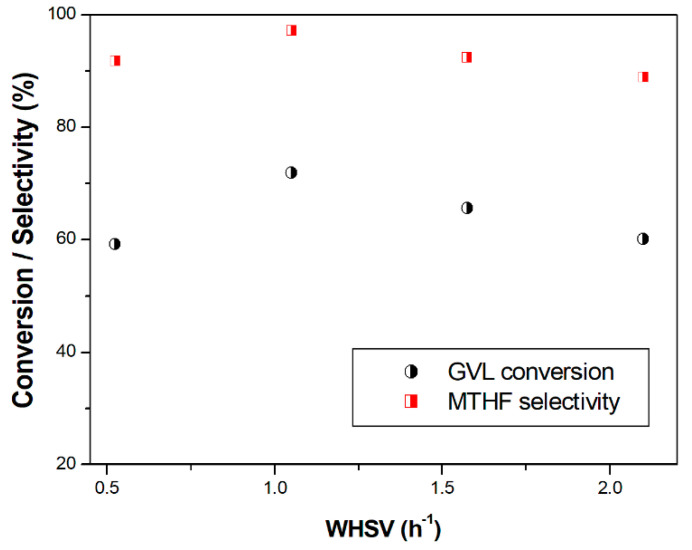
Influence of WHSV on the catalytic performance of 5 wt% Cu/SiO_2_. Reaction conditions at various WHSV: 10 wt% GVL of reaction feed, 0.4 g of 5 wt% Cu/SiO_2_ catalyst, 260 °C reaction temperature.

The effects of WHSV (0.525, 1.05, 1.575 and 2.10 h^−1^ with respect to GVL) on the GVL hydrogenation procedure were examined using a 5 wt% Cu/SiO_2_ catalyst. Figure 10 presents the WHSV values calculated while keeping the catalyst’s weight constant by increasing the GVL input flow from 0.25 mL/h (WHSV = 0.525 h^−1^) to 1.0 mL/h (WHSV = 2.10 h^−1^). Due to an increase in the feed’s contact time with the catalyst surface, the greatest GVL conversion and MTHF selectivity were seen at saturated WHSV (1.05 h^−1^). Because the feed’s retention time fell, along with the catalyst’s surface area, as WHSV increased from 1.05 to 2.1 h^−1^, the GVL conversion and MTHF selectivity also reduced.

#### 3.2.5. Influence of Time-on-Stream

**Figure 11:**
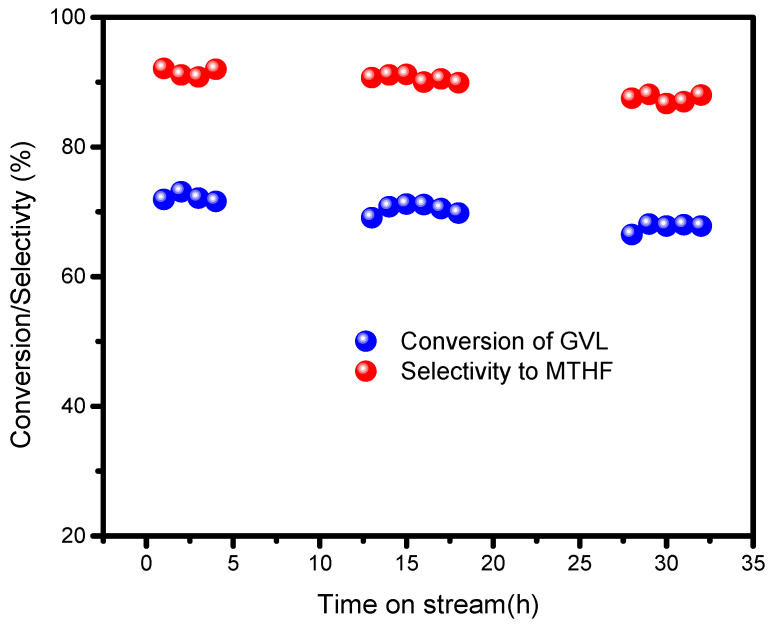
Time on stream study of the 5 wt% Cu/SiO_2_ catalyst.

A 33 h investigation into the impact of stream time on conversion and selectivity using a 5 wt% Cu/SiO_2_ catalyst at 260 °C is depicted in Figure 11. This catalyst exhibits stable catalytic activity towards both the GVL conversion and MTHF selectivity. No change was observed, even at the start of the 33 h investigation. Hence, this catalyst does not show any catalytic deactivation for this reaction, probably due to the mild reaction conditions and the highly suppressed coke formation and active sites sintering. Moreover, metal leaching did not appear which might also be accounted for by the lower metal (5 wt% Cu) loading of the SiO_2_ support.

#### 3.2.6. Deactivation Studies

**Figure 12:**
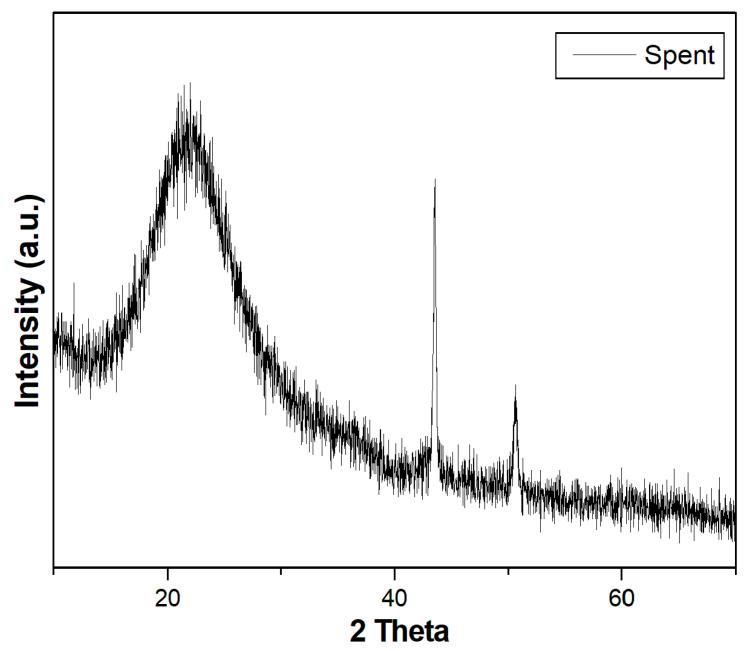
XRD pattern of spent 5 wt% Cu/SiO_2_ catalysts.

To further investigate catalyst stability, the spent 5 wt% Cu/SiO_2_ catalyst was analyzed by XRD and the results are shown in Figure 12. It can be seen from Figure 2 that the reduced 5 wt% Cu/SiO_2_ catalyst has two primary Cu metallic-related diffraction peaks at 2*θ* = 43.5° and 50.5°. The XRD pattern of the spent catalyst displayed a number of intense peaks associated with CuO and Cu_2_O species. CuO peaks in the used catalyst may result from highly distributed Cu phase oxidation interacting with the support, and a rise in the strength of the CuO peaks may be caused by an increase in the size of the CuO species crystallites. As a result, the reductive regeneration of a used catalyst during thermal conversion, while hydrogen flow was present, improved the recovery of catalytic activity.

## 4. Conclusions

The fabrication of catalysts with varying Cu loadings (2–20 wt%) with SiO_2_ support by wet-impregnation is an inexpensive and efficient method, following calcination. The prepared catalysts were characterized by physicochemical measurements. The results showed that the best catalyst had a surface area of 194 m^2^ g^−1^, a pore volume of 0.2199 cm^3^ g^−1^, a Cu metal area of 132 m^2^ g^−1^, a dispersion of Cu of 20.6%, and 133 µmol g^−1^ of acidic sites. Various reaction conditions, including the effect of Cu loading, temperature, time and catalyst concentration were optimized in the reaction protocol for the selective vapour-phase conversion of GVL to MTHF. A 5 wt% Cu/SiO_2_ catalyst exhibited high catalytic activity, selectivity and stability due to the high dispersion of Cu on the support, the surface area, the acidic sites and the H_2_ activation ability. This work proposes an efficient route for the vapour-phase catalytic selective hydrogenation of biomass resources.

## Figures and Tables

**Table 1:** Textural properties of various Cu/SiO_2_ catalysts.

Cu Loading (wt%)	Total Pore Volume (cm^3^ g^−1^)	Average Pore Size (nm)	BET Surface Area (m^2^ g^−1^)
2	0.3421	5.99	228
5	0.2199	4.53	194
10	0.1745	4.56	153
15	0.1661	4.62	144
20	0.1309	4.31	133

**Table 2:** H_2_ consumption and reaction temperature of the various Cu/SiO_2_ Catalysts from H_2_-TPR profiles.

Cu Loading (wt%)	H_2_ Consumption (µmol g^−1^)		Total H_2_ Consumption (µmol g^−1^)	Peak Temperture T _max_ (°C)	
	Surface Reduction (S_r_)	Bulk Reduction (B_r_)		S_r_	B_r_
2	195	-	195	282	-
5	432	230	662	275	383
10	365	1024	1389	269	387
15	2178	-	2178	255	-
20	2834	-	2834	246	-

**Table 3:** Percentage dispersion, average particle size, metal surface area, and H_2_ uptake of various Cu/SiO_2_ catalysts from N_2_O decomposition.

Catalyst Loading (wt%)	Cu-Dispersion (%)	Specific Copper Surface Area (m^2^ g^−1^_Cu_)	H_2_ Uptake (µmol g^−1^)	Average Particle Size (nm)
2	18.8	120	32	5.55
5	20.6	132	81	5.11
10	11.5	76	90	8.97
15	8.01	52	95	13.2
20	5.22	34	81	20.7

**Table 4:** NH_3_-TPD of SBA-15 and different Cu/SiO_2_ samples.

Cu Loading (wt%)	NH_3_ Desorbed (µmol g^−1^)		Total NH_3_ Desorbed (µmol g^−1^)	Peak Temperature T _max_ (°C)	
	Weak	Medium		Weak	Medium
2	15	108	123	71	297
5	14	119	133	70	299
10	8	88	96	67	292
15	7	56	63	67	285
20	6	31	37	68	256

**Table 5:** Hydrogenation of GVL to 2-MTHF over various Cu/SiO_2_ catalysts with various Cu loadings.

Catalyst (% Cu Loading)	GVL Conv. (%) ^a^	Selectivity (%) ^b^		
		MTHF	PDO	Others
2	15.4	96.0	1.9	2.1
5	21.6	98.1	0.3	1.6
10	17.1	90.2	7.3	2.4
15	14.7	88.2	9.1	3.7
20	9.4	72.9	22.7	4.3

^a^ Reaction conditions: feed rate of GVL: 0.50 mL h^−1^; weight of Cu/SiO_2_ catalyst 0.4 g; reaction temperature: 200 °C; H_2_ gauge pressure: 0.1 MPa; H_2_ flow rate 50 cm^3^ min^−1^. ^b^ MTHF: 2-methyltetrahydrofuran; PDO: 1,4-pentanediol.

**Table 6:** Comparison between catalytic activity with catalyst properties in different Cu/SiO_2_ catalysts.

Cu Loading (wt%)	Specific Copper Surface Area (m^2^ g^−1^_Cu_)	Average Particle Size of Cu (nm)	Conversion of GVL (%)	Selectivity of MTHF (%)
2	120	5.55	15.4	96.0
5	132	5.11	21.6	98.1
10	76	8.97	17.1	90.2
15	52	13.2	14.7	88.2
20	34	20.7	9.4	72.9

**Table 7:** Hydrogenation of GVL to 2-MTHF over 5% Cu/SiO_2_ catalysts over various temperatures.

Reaction Temperature (°C)	GVL Conv. (%) ^a^	Selectivity (%)		
		MTHF	PDO	Others
200	21.6	98.1	0.3	1.6
220	35	98.4	0.5	1.1
240	43	95.6	2.3	2.1
260	71.9	97.2	1.1	1.7
280	82.1	75.1	20.6	4.3
300	96.7	83.9	13.9	5.2

^a^ Reaction conditions: feed rate of GVL: 0.50 mL h−1; weight of Cu/SiO2 catalyst: 0.4 g; H2 gauge pressure: 0.1 MPa; H2 flow rate: 50 cm3 min^−1^.

## Data Availability

Data are contained within the article.
